# Effectiveness of arts interventions to reduce mental-health-related stigma among youth: a systematic review and meta-analysis

**DOI:** 10.1186/s12888-021-03350-8

**Published:** 2021-07-22

**Authors:** Shivani Mathur Gaiha, Tatiana Taylor Salisbury, Shamaila Usmani, Mirja Koschorke, Usha Raman, Mark Petticrew

**Affiliations:** 1 Indian Institute of Public Health- Hyderabad, Public Health Foundation of India, Hyderabad, India; 2 Department of Public Health, Environments and Society, Faculty of Public Health and Policy, London School of Hygiene and Tropical Medicine, London, UK; 3 Department of Pediatrics, Division of Adolescent Medicine, Stanford School of Medicine, Stanford University, Palo Alto, USA; 4 Health Service and Population Research Department, Institute of Psychiatry, Psychology and Neuroscience, King’s College, London, UK; 5 Centre for Global Mental Health, London School of Hygiene and Tropical Medicine, London, UK; 6 Department of Communication, Sarojini Naidu School of Arts & Communication, University of Hyderabad, Hyderabad, India

**Keywords:** Mental health, Youth, Stigma, Art, Systematic review, Meta-analysis, Performing, Film, Role-play, Theatre

## Abstract

**Background:**

Educational interventions engage youth using visual, literary and performing arts to combat stigma associated with mental health problems. However, it remains unknown whether arts interventions are effective in reducing mental-health-related stigma among youth and if so, then which specific art forms, duration and stigma-related components in content are successful.

**Methods:**

We searched 13 databases, including PubMed, Medline, Global Health, EMBASE, ADOLEC, Social Policy and Practice, Database of Promoting Health Effectiveness Reviews (DoPHER), Trials Register of Promoting Health Interventions (TRoPHI), EPPI-Centre database of health promotion research (Bibliomap), Web of Science, PsycINFO, Cochrane and Scopus for studies involving arts interventions aimed at reducing any or all components of mental-health-related stigma among youth (10–24-year-olds). Risk of bias was assessed using the Effective Public Health Practice Project (EPHPP) Quality Assessment Tool for Quantitative Studies. Data were extracted into tables and analysed using RevMan 5.3.5.

**Results:**

Fifty-seven studies met our inclusion criteria (*n* = 41,621). Interventions using multiple art forms are effective in improving behaviour towards people with mental health problems to a small effect (effect size = 0.28, 95%CI 0.08–0.48; *p* = 0.007) No studies reported negative outcomes or unintended harms. Among studies using specific art forms, we observed high heterogeneity among intervention studies using theatre, multiple art forms, film and role play. Data in this review are inconclusive about the use of single versus multiple sessions and whether including all stigma components of knowledge, attitude and behaviour as intervention content are more effective relative to studies focused on these stigma components, individually. Common challenges faced by school-based arts interventions included lack of buy-in from school administrators and low engagement. No studies were reported from low- and middle-income countries.

**Conclusion:**

Arts interventions are effective in reducing mental-health-related stigma to a small effect. Interventions that employ multiple art forms together compared to studies employing film, theatre or role play are likely more effective in reducing mental-health-related stigma.

**Supplementary Information:**

The online version contains supplementary material available at 10.1186/s12888-021-03350-8.

## Background

Stigma or a negative disposition towards mental ill-health and people with mental health problems is a widely recognized barrier in help-seeking for mental health problems [1]. Public stigma, consists of ‘problems of knowledge (ignorance), problems of attitude (prejudice), and problems of behaviour (discrimination).’ [2] Such stigma especially inhibits help-seeking by youth due to their inability to recognize mental health problems, difficulty in talking about their problems for fear of peer pressure and a negative perception of people with mental health problems as dependent, which clashes with their desire to be self-reliant [3]. Thus, although an estimated 10–20% of youth aged 10–24 years suffer from mental health problems, [4] 63–86% of all mental health problems that require a diagnosis generally go undetected [5]. Therefore, interventions targeting non-clinical youth groups to reduce mental-health-related stigma may promote youth help-seeking behaviour and ultimately address unattended mental health needs.

Most anti-stigma interventions and/or campaigns have been conceptualized using knowledge-attitude-behavior paradigm [6]. Knowledge is defined as information an individual perceives about mental health as a function of memory and stereotyping (related to, for e.g., treatment efficacy, symptom recognition, help-seeking, and employment), attitude is defined as perceptions or views towards people with mental disorders or about mental disorders (related to negative attitudes, for e.g., desiring social control and social distance), and behavior as intended or actual discriminatory actions towards people with mental health problems (related to, for e.g. social exclusion, which may contribute to status loss or human rights violations of someone living with a mental health problem) [2, 7–9]. Further, effective strategies in anti-stigma interventions include education, social contact (interaction with a person who suffers from a mental health problem) and protest [10]. In addition, effective interventions are often locally tailored, perceived as credible and of a longer duration [11]. In school-based settings, experiential learning (learning through reflection on doing), empathy building, interactive and prolonged exposure to anti-stigma content is likely effective [12, 13]. Overall, systematic reviews of anti-stigma intervention studies report that in the long term and among youth, educational interventions are likely more effective than social contact interventions in reducing stigma with moderate effect [14–16]. Among the approaches used, educational interventions have employed a variety of visual, literary and performing arts to improve relatability, interactivity and engagement.

Art is broadly defined as any means for expression of individual and social values, through concrete and artistic activities and processes [17]. Further per Dewey’s conceptualisation of art, arts interventions may communicate moral purpose or education [18] or explain experiences of one’s daily emotional and rational world [19]. The evidence for arts-based educational interventions is generally limited, despite its documented emotional and visceral effects [20]. Despite multiple, relevant systematic reviews, uncertainties remain regarding the overall effectiveness of arts-based interventions in reducing mental-health-related stigma and relative effectiveness of interventions employing different art forms, varying durations and conceptualizations of stigma. A review of 22 studies evaluating the impact of mass media interventions including film, photographs, radio and comics attributed reduced prejudice (attitude) for mental health problems to creative and artistic content [21]. The majority (86%) of studies in this review focused on student populations. Other reviews of studies among 11–18 year olds using creative activities such as music, dance, singing, drama and visual arts [22] and performing arts, [23] indicate that arts-based interventions improve knowledge, another component of mental-health-related stigma. As some reviews are focused on educational versus social contact-based interventions, [14–16] they do not focus on the distinguishing role of arts-based elements in achieving impact nor suggest the relative impact from using role play, theatre, film compared to other art forms.

Previous studies show that arts-based interventions have the potential to reduce mental-health-related stigma as they have improved individual components of such stigma, i.e. attitude and knowledge. However, little is known about the effectiveness of arts-based interventions in reducing overall mental-health-related stigma among youth, and whether interventions using specific art forms, duration and content on all stigma components of knowledge, attitude and behavior are more effective in reducing such stigma compared to individual components. The objectives of this study are to: (a) assess the effectiveness of arts-based interventions to reduce stigma associated with mental health among youth; (b) assess effectiveness of arts-based interventions by their duration; (c) assess whether a comprehensive approach to stigma is more effective than a focus on individual stigma components; and (d) identify barriers and facilitators in implementation of arts-based interventions and the role of implementation in building participant engagement and ultimately influencing how effective such interventions are in reducing stigma.

## Method

### Eligibility criteria

Studies will be included in the review if they contain:

- Interventions using any form of art or creative expression or storytelling as a key method were included. Such art forms include (1) using words in literary art (in stories, poetry, creative writing, essays and other forms), and through creation of physical objects and experiences, through (2) visual art (drawing, painting, sculpture, crafts, pottery, installation), and (3) performing art (theatre or dramatic improvisation or role-play, dance, puppetry, music, stand-up comedy, folk dance-drama). In this review, participants in included arts interventions should either be exposed to art (e.g., as an active observer/audience interpreting and responding to scenarios in a theatre production) or create their own art (e.g., as generating thought, meaning, aesthetic or object/s).
- Interventions delivered to youth aged 10–24 years.
- Outcomes related to at least one component of mental-health-related public stigma (three components outlined by Thornicroft et al. as problems of knowledge, attitude and behavior). Based on the literature any of these factors individually or in combination with one another contribute towards such stigma.
- Qualitative, quantitative and mixed methods research. Study designs include controlled studies, including randomised trials, controlled clinical trials, cohort analytic studies and case-control studies. Pre-and post-studies with a single cohort and post-test only studies, qualitative and mixed methods studies were also included. Conference abstracts and case studies were included to capture all interventions. Mixed methods studies were defined as studies which involved “sequential or simultaneous use of both qualitative and quantitative data collection and/or data analysis techniques.” [24]

Studies were excluded from the review if they met one of the following criteria:

- Target clinical, high-risk or at-risk populations (youth with mental disorders, including outpatients, in schools for special needs, in prisons, foster homes/ shelters and conflict zones or exposed to violence) or caregivers as these groups have unique personal experiences that might distinguish them from the general population.
- Use mass media (newspapers, television and radio programmes, advertising, popular culture, cinema and songs, social media, blogs and other Internet or mobile phone).
- Combine art with other strategies, where the effect of art is not separately reported.
- Focus on intimate partner violence, sexual violence and gender-based violence, cyberbullying and domestic abuse.

### Search strategy

The broad categories of terms used included art; mental health disorders/conditions; youth; and stigma (see Supplementary Table 1 for exact search terms used). The search strategy included Medical Subject Headings (MeSH) terms, where appropriate. Thirteen academic databases were searched: PubMed, Medline, Global Health, EMBASE, ADOLEC, Social Policy and Practice, Database of Promoting Health Effectiveness Reviews (DoPHER), Trials Register of Promoting Health Interventions (TRoPHI), EPPI-Centre database of health promotion research (Bibliomap), Web of Science, PsycINFO, Cochrane trials and database of systematic reviews and Scopus. Additional articles were searched using Google Scholar. The search was not limited by publication dates, countries or languages. This initial search for inclusion of papers was completed on 19 July 2018. From 28 March 2021 to 3 April 2021, the search was updated in all databases, except Global Health, Social Policy and Practice and Scopus (which the first author could no longer access). If two or more articles on the same intervention and target population were found, the most relevant article was retained for analysis. The Preferred Reporting Items for Systematic reviews and Meta-analyses (PRISMA) guidelines were used to report updated study findings (see Supplementary Table 2 for checklist) [25].

### Data extraction

All titles and abstracts were assessed by a single reviewer (SMG). A second reviewer (SU) assessed 10% of all titles and abstracts to confirm accuracy of inclusion. The updated search was conducted by the first author and 895 additional articles were retrieved. Using the Quality Assessment Tool for Quantitative Studies developed by the Effective Public Health Practice Project (EPHPP), [26] a framework for data extraction was developed. The framework captured additional data on intervention characteristics and study design, related to review objectives. Full-text articles were independently assessed as per the EPHPP framework and data were entered in to tables by a single reviewer (SMG). The second reviewer assessed all full-text articles and cross-checked data in the framework. Discussion between reviewers compared quality ratings and key findings. Where consensus was not reached, a third reviewer (MP) was consulted.

### Summary measures

The main study outcome was mental-health-related public stigma, which is composed of three components: knowledge, attitude and behaviour. Measures of these components include means and standard deviations, difference between means and level of significance (*p*-value).

### Synthesis and reporting of results

Demographic information of participants and qualitative themes were compiled in a narrative form. Firstly, means, standard deviations and sample sizes were pooled for each stigma-component/outcome for all studies, followed by art form or intervention type, to assess whether the type of intervention was responsible for a difference in outcomes. Change in stigma was plotted by pooling study-wise difference of means and standard deviations per component of stigma among studies with a study design rating of 1 or 2 per the EPHPP component ratings. As an illustration, for the behavior component of stigma we pooled mean differences from the Social Distance scale (a common proxy measure for behavioral intent) [27] and the Reported and Intended Behavior Scale [28]. If a study reported on multiple items within each stigma component, then the item with the lowest (stigmatising) mean score change was included. We calculated the mean score change from data available in the study text and tables, wherever available. Change in outcomes related to stigma (knowledge, attitude and actual or intended behaviour) were pooled by intervention type or art form, i.e. multiple art forms, film, theatre and role play. Next, a post-hoc sub-group analysis of data was conducted by intervention duration (single versus multiple sessions). Finally, studies which took a comprehensive approach to stigma (measured knowledge, attitude and behaviour components, together and likely also included content addressing each of these components) were pooled for their the impact on individual stigma components. These analyses were presented alongside pooled studies measuring individual stigma components such as knowledge or behaviour alone. This was done in order to assess whether a comprehensive approach leads to better outcomes within each stigma component. Meta-analysis, where appropriate, was conducted using Review Manager software (Version 5.3.5) [29]. Heterogeneity of studies was assessed through I^2^ values> 0, and random-effects models were generated to calculate the effect size on stigma. A random-effects model with standardized mean differences was preferred as study populations and locations, recruitment processes, points of time for implementation and assessment measures varied. Narrative synthesis was used to collate findings regarding barriers and facilitators in reducing stigma.

## Results

**Fig. 1:**
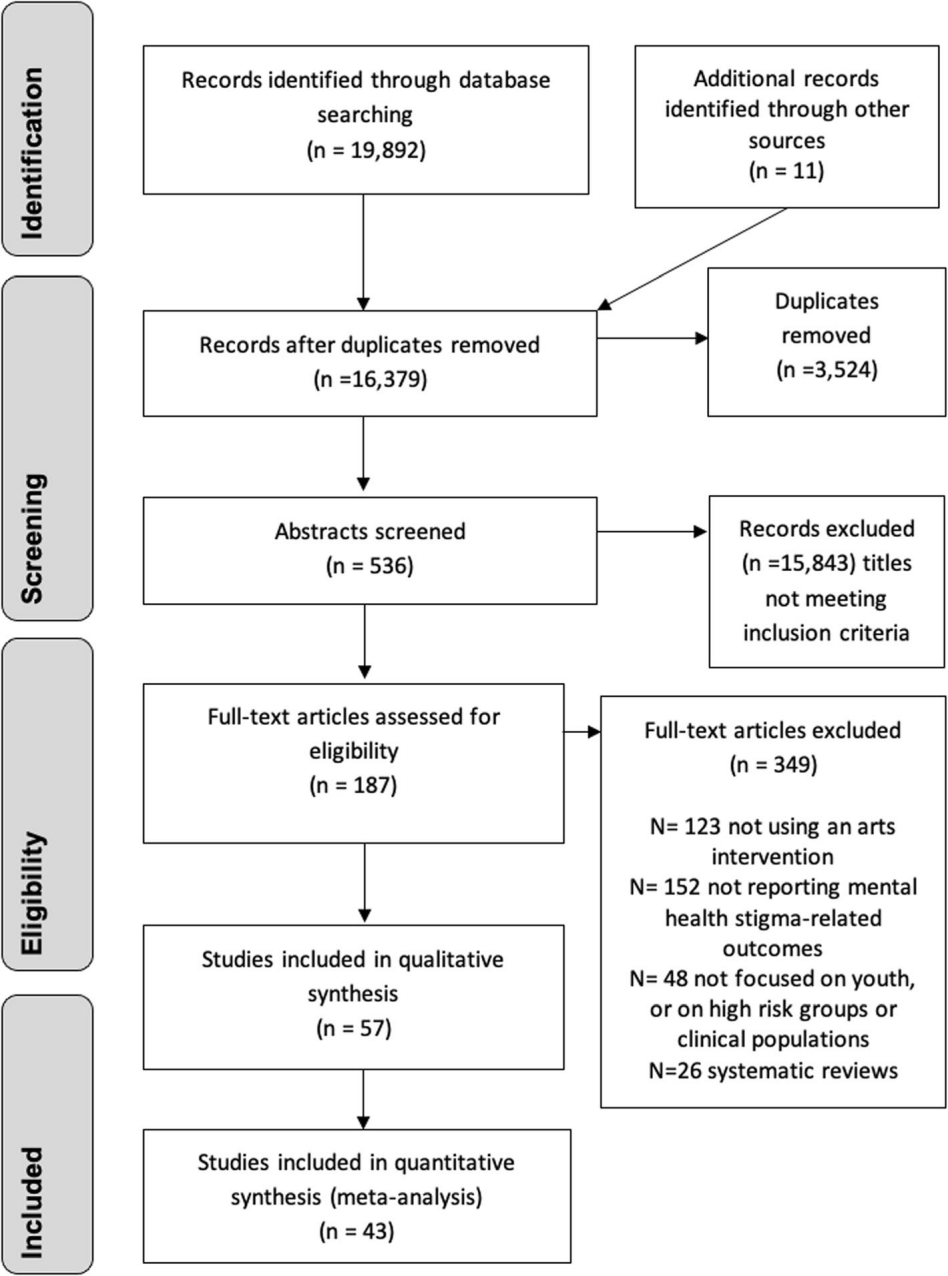
PRISMA flow chart

The search produced 19,892 articles, of which 187 articles were identified for full-text review (Fig. 1). Of these, 132 were either contextual articles without an arts intervention, epidemiological studies assessing impact from exposure to media, reviews on related aspects of stigma or youth or studies that did not meet the inclusion criteria. Finally, 57 studies (53 full-text articles and four conference abstracts) were included in this systematic review.

### Study characteristics

Of the 57 included studies, 43 quantitative studies, [30–72] six qualitative studies [73–78] and eight mixed methods studies [79–86] were identified. Data from 57 studies (by intervention type) on sample size, participant profile, study design, intervention description, duration and frequency, number of follow-ups, and outcomes related to knowledge (K), attitude (A) and actual/ intended behaviour (B) are summarised in Table 1. Quantitative studies reached 26,634 youth and eight mixed methods studies reached 14,021. Qualitative studies engaged 966 youth, however the number of participants is unclear in two studies [73, 87].

**Table 1:** Summary of study characteristics and outcomes

**A. Studies using theatre interventions [11 studies]**												
**Author/s, Year**	**Country**	**Sample size**	**Age [mean (SD)/ range]**	**Participant profile**	**Study Design**	**Intervention description**	**Duration (frequency)**	**Number of follow-ups (times)**	**Description of change in all stigma dimensions (knowledge (K), attitude (A) and behaviour (B)**	**Change in stigma** *(Difference of means)*		
										**K**	**A**	**B**
Faigin DA & Stein C, 2008 [38]	USA	303	19/ 18–40	College students (Health professionals in-training)	Controlled clinical trial	Live and video-taped theatrical performance	70 min (once)	2 (immediately post and after 1 month)	More benevolent attitudes. Students gained more knowledge through lesson plans than theatre.	–	0.18	0.15
Gliksman DL et al., 1983 [41]	Canada	716	14–18^a^	School students	Cohort analytic (two group pre and post)	BOOZE- series of five skits and theatre-based lesson plan	5 h (Not Reported (NR))	1 (1 week post)	Significant change in attitude to alcohol use between groups (*p* < 0.03). No change in attitude towards alcohol abuse/ alcohol education. Live theatre stimulated thought and discussion on effects of drugs (53% learnt something new).	–	–	–
Harding C et al., 1996 [74]	USA	580	14–18	School students	Qualitative research	Captain Clean- Professional musical play (18 performances)	30 mins (once)	1 (2 weeks post)	94 individual counselling requests (increase); 60% would stand up against drugs; “true friends would not involve them in drug-related activities”	–	–	–
Jones N et al., 2014 [47]	UK	594	75% < 30 years	Miltary personnel	Cohort analytic (two group pre and post)	Stand-up comedy show	(once)	2 (immediately post and after 3 months)	No significant effect on RIBS after controlling for baseline score; but borderline significant effect at follow-up. Significant change in knowledge between baseline and posttest, but no significant effect at follow up (*p* = 0.15).	0.37	0.01	0.3
Pitre N et al., 2007 [57]	Canada	185	8–12	School students	Controlled clinical trial	Puppet show on schizophrenia, dementia and anxiety/ depression	45 min (once)	1 (day aft48intervention for experimental group and 2 weeks after for control group)	Significant change in restrictive attitude towards people with mental health problems. Experimental group preferred significantly lower Separatism (*p* < 0.01), and Stigmatization (*p* < 0.025). However, stereotyping was not significantly different.	–	–	–
Roberts G et al., 2007 [60]	UK	2500	14–22	School students	Cohort (one group pre-post design)	71 performances	4 h in 3 weeks (NR)	2 (1–2 weeks post and after 6 months with a subgroup)	Significant increase in student willing to seek help (5.4% change from baseline to posttest and 1.7% change from baseline at follow up). Significant positive change in beliefs about treatment, dangerousness and difficulty in talking to people with mental health problems. Participants showed significant gain in knowledge about where to go for help, including clinical options.	–	–	–
Rowe N et al., 2013 [75]	Malaysia	5	20–22	College students (Theatre major)	Qualitative research	Collaborative theatre with people living with mental health problems	8 months (NR)	NR details of follow up after the project	Process helped to acknowledge labels. Change from initial hypersensitivity, cautiousness and awkwardness or pity to – ‘I want to make people aware that difference is not dangerous.’- Normal, not taboo, ordinary relationship and comfortable were words used to describe social contact. Participants understood that depression, anxiety, panic and stress are related to mental health.	–	–	–
Safer LA & Harding CG., 1993 [61]	USA	278	10–12	School students	Cohort analytic (two group pre and post)	Captain Cle–n - live musical play and role play	30 min (NR)	1 (2–3 weeks posttest)	19% students requested counselling. More positive attitudes at post-test with no change in control group.	–	–	–
Starkey F & Orme J., 2001 [84]	UK	285	10–11	School students	Mixed method (based on a one group pre-post design)	Interactive drama production and workshop	One day (once)	1 (4 weeks post)	‘A person who lost a bag of drugs is not silly/ stupid (3.5% change; *p* < 0.01). Likely to call the police if they found drugs and were able to identify names of drugs.	–	–	–
Twardzicki M., 2008 [65]	UK	67	16–19	College students (general major)	Cohort (one group pre-post design)	Collaborative art through social contact	3 years (3 half days + performance)	0	Students expressed willingness to help people with mental health problems or visit a relevant organisation. 18/43 participants showed a more positive attitude. 30/43 showed improved understanding of mental health.	–	–	–
Welch TR & Welch M., 2008 [77]	Canada	80	NR	College students (Health professionals in-training)	Qualitative research	Bearing Witness- play about an abuse survivor (3 performances and a staged production)	NR (once)	1 (after 4 months)	Participants related at a cognitive and emotional level. Personal stories aroused empathy. Ability to engage and yet ‘step away’ was important. Participants’ gained clinical knowledge.	–	–	–
**B. Studies using multiple art forms [23 studies]**												
**Author/s, Year**	**Country**	**Sample size**	**Age [mean (SD)/range]**	**Participant profile**	**Study Design**	**Brief intervention description**	**Intervention Duration (frequency)**	**Number of follow-ups (times)**	**Description of change in all stigma dimensions (knowledge (K), attitude (A) and behaviour (B)**	**Change in stigma** *(Difference of means between experimental and control groups)*		
										**K**	**A**	**B**
Chan HV & Pervanas HC., 2014 [32]	USA	24	11–12	College students (Health professionals in-training)	Post-test only for one group	Video skit and interactive visual material	NR (once)	NA	Raised awareness of drug and alcohol abuse (no specific changes)	–	–	–
Duryea E et al., 1984 [36]	USA	155	14–15^a^	School students	Cohort (one group pre-post design)	Film, role play, slide show	6 school days (1 h per day)	2 (1 week post and after 6 months)	Ability to refute pro-drinking arguments by treatment (*p* < 0.005) and time (*p* < 0.001). Significant increase in ability to answer multiple choice questions on alcohol (*p* < 0.001).	1.82	0.27	–
Gilfoy K & Young A, 2001 [73]	UK	NR	13–21	Youth in a community setting	Qualitative research	Co-creation music, documentary and visual arts	2 weeks during summer (NR)	0	Focus on awareness, not changing views. Raising awareness within the peer group explored.	–	–	–
Stevens V et al., 2000 [62]	Belgium	1465	13–16	School students	Cohort (one group pre-post design)	Film and role play	6.6 h or 400 min (NR)	2 (after 6 months of baseline and after 12 months)	Most students reported a negative attitude towards bullying behaviours, but few of them intervened.	–	0.05	0.22
Jones S et al., 2011 [48]	UK	109	14–15^a^	School students	Post-test only for one group	Video, word association and role pay	50 min (once)	1 (immediately post)	Dispelled stereotypes that people with mental illness do not look scary among 25% participants. About 40% gained knowledge that mental illness is common, 20% learnt about anxiety, depression and < 1% reported learning about where to seek help.	–	–	–
Kassam A et al., 2011 [50]	UK	65	22.8(4.4)	College students (Health professionals in-training)	Controlled clinical trial	Presentation and role play	1 h 30 mins	1 (1 week post)	Factual knowledge improved significantly (p < 0.001). However, there was no change in attitudes and behaviour.	1.4	0.6	–
Marques Filho et al., 2007 [81]	Brazil	94	20–25^a^	College students (Health professionals in-training)	Mixed methods	Mind fingers song	NR (NR)	0	Reflection group using lyrics helped in minimization of resistances to do with talking about drug use, attitudes about understanding psychoactive substances, contemplating use and abstinence, facilitating the transmission of knowledge to students.	–	–	–
Kalafat J & Elias M., 1994 [49]	USA	253	15–16^a^	School students	Controlled clinical trial (Solomon group design)	Role play, video and didactic session on how to respond	45 min (3 sessions)	1 (immediately post)	Significant overall group effects on knowledge (p < 0.001), attitude (p < 0.03) and behaviour (*p* < 0.002). Participants more likely to take effective action for a troubled peer/ self: call a hotline (*p* < 0.05) or take a friend’s advice (*p* < 0.05). Participants more likely to disagree with negative statements about seeking help and intervening with suicidal individuals and with stereotypes that suicide runs in families (males commit it more often and people who talk about it do not do it).	–	–	–
Mora M et al., 2015 [54]	Spain	200	12–15	School students	Controlled clinical trial	Interactive multimedia and performing “Teen Spirit,” a professionally scripted play	120 min (10 sessions)	3 (post-test after 1 month, and after 5 and 13 months)	Change in attitude towards eating disorders through reduced thin-ideal internalization.	–	–	–
Paukste E & Harris N., 2015 [82]	Australia	18	14–18	School students	Mixed methods	Creative workshops and educational sessions	1–2 h (7 weeks)	1 (final week of the workshop)	Understanding of risk and changed perspectives on alcohol, tobacco and other drugs	–	–	–
Altindag AM et al., 2006 [30]	Turkey	60	19–25^a^	College students (Health professionals in-training)	Cohort analytic (two group pre and post)	Education (2 h lecture), social contact and film on schizophrenia (A beautiful mind)	One day (once)	1 (1 month post)	Attitudes towards social distance towards people living with Schizophrenia and willingness to work with a person living with Schizophrenia	–	–	–
Friedrich B et al., 2013 [40]	UK	1452	23.5	College students (Health professionals in-training)	RCT	Time to Change END intervention; short lecture, professional role play and feedback	Three years (NR)	2 (immediately post and after 6-months)	Participants had a significantly greater improvement in intended behaviour, attitude (2/3 item–s – easy to recognise a person with MI and frightening to have people with MI in the neighbourhood) and knowledge than the control group. While knowledge changed significantly at follow up, behaviour showed no change and attitude changed only for one item.	–	–	–
Van Schoiack-Edstrom, L et al., 2002 [66]	USA	714	10–14^a^	School students	Cohort analytic (two group pre and post)	Videotaped vignettes, reading newspaper stories and role play	One semester (15 lessons; 8 lessons)	1 (Between 1 and 5 weeks post)	Reduced endorsement of verbal derogation and social exclusion in relation to physical aggression	–	–	–
Essler V et al., 2006 [37]	UK	104	13–14	School students	Cohort (one group pre-post design)	Professional theatre, quiz, drama and games	NR (NR)	1 (1 month post“)	“stay friends”-; risk of violence by mental health persons reduced p = 0.015) increase in median scores; *p* = 0.015 (no comparison or baselines data); significant increase in knowledge of incidence of symptoms	–	–	–
Wasserman C et al., 2012 [86]	11 European countries	12,395	14.9 (0.9)/ 14–16	School students	Mixed methods (based on a RCT)	Graphic booklet, role play and posters	5 h in four weeks (weekly)	2 (after 3 months and after 12 months)	desire to help persons in need; increased general mental health awareness and self-recognition	–	–	–
Woodside et al., 1997 [69]	USA	588	11–15^a^	School students	Cohort analytic (two group pre and post)	The Images Within– Visual art, learner’s guide and brochures	NR (once)	1 (immediately post)	Increase in student referrals from 50 to 113%. Significant improvement in attitude to helping a friend from an alcoholic family between treatment-control (*p* < 0.001) and between pre-test- post-test (*p* < 0.009). Knowledge about alcohol improved by 15.2% (change in score), its effects on the family improved by 12.7% and significant changes between treatment-control (*p* < 0.001) and pre-test-posttest (p < 0.001) regarding the need for help.	–	–	–
Rabak-Wagener J et al., 1998 [58]	USA	105	18–23	College students (Health professionals in-training)	Cohort analytic (two group pre and post)	Slim Hopes video, advertisements and magazine photographs, collage-making and discussion	1 h 35 min in 4 sessions (weekly)	1 (3 weeks after pre-test)	High agreement on beliefs and behaviours related to body image.	–	−2.93	–
Watson R & Vaughn LM, 2006 [67]	USA	54	19.21 (1.67)/ 18–25	Female college students (general major)	Cohort analytic (two group pre and post)	Video, popular magazine images, role play, homework and discussion	1.5 h and 1.5 h × 4 weeks (weekly)	1 (immediately post-test)	A long-term media literacy intervention was more effective at decreasing body dissatisfaction than a similar short term. Video only, short-term interventions did not have an effect on awareness. Change in awareness in pre-test to post-test was observed in the long term condition, t(14, 15) =4.617, *p* < 0.01.	–	–	–
Stuart H, 2006 [63]	Canada	571	13–18^a^	School students	Cohort (one group pre-post design)	Video (20 mins), role play and discussion	NR (Once)	1 (after 3 weeks)	14% increase in number of students who were not afraid to talk to someone who had schizophrenia. Students were about 4 times more likely to achieve a high knowledge score (80% or greater) but only about twice as likely to achieve a high distance score. Improvement in knowledge at post-test (p < 0.001).	–	–	–
Kusel A, 1999 [53]	USA	172	9–12	School students	RCT	Videos, magazine review and discussion	Two days	1 (1 month after pre-test)	Significant decrease in internalization of body stereotypes over time and between treatment-control. Findings show that young girls were able to critically analyse portrayals of body types in the media.	–	–	–
Pervanas, et al., 2014 [56]	USA	24	11–17	Boys and girls clubs	Cohort (one group pre-post design)	Video and role play on substance abuse	Single session	1 (immediately post)	Improved knowledge about safety and dangers of taking drugs and getting sick.	–	–	–
Gubner, J. et al., 2020 [78]	USA	52	–	Undergraduate college students (54% majoring in Health Sciences)	Qualitative research	Music, filmmaking, reflective essay writing and service at local dementia care settings	Three consecutive semesters	Multiple times; throughout the course duration	Music and filmmaking enable students to share individual stories about dementia and reflective writing supports students to gain self-awareness related to dementia stigma by processing experiences.	–	–	–
Hui, C.L.M. et al., 2018 [44]	Hong Kong	4520	12–17	Secondary school students	Cohort (one group pre-post design)	“School Tour” – drama and presentation on psychosis; exercises and yoga	1 h	1 (immediately post-test)	Significant improvements in knowledge and attitude towards psychosis between pre-test and post-test.	–	0.1	–
**C. Studies using film [17 studies]**												
**Author/s, Year**	**Country**	**Sample size**	**Age [mean (SD)/range]**	**Participant profile**	**Study Design**	**Brief intervention description**	**Intervention Duration (frequency)**	**Number of follow-ups (times)**	**Description of change in all stigma dimensions (knowledge (K), attitude (A) and behaviour (B)**	**Change in stigma** *(Difference of means between experimental and control groups)*		
										K	A	B
Aseltine R. et al., 2004 [31]	USA	2100	14–18	School students	RCT	Video and discussion	2 days (over two months)	1 (immediately post-test)	Help-seeking behaviour did not change significantly between treatment and control group. Participants showed more adaptive attitudes and greater knowledge relate to depression and suicide (effect size = 0.35, p = 0.007).	0.69	0.25	–
Clement S et al., 2012 [34]	UK	216	23.9 (6.9)	College students (Health professionals in-training)	RCT	DVD and live social contact	71 min (once)	2 (immediately post and after 4 months)	Participants who watched the DVD had better attitude and behaviour scores than the lecture group (*p* = 0.004), the latter difference maintained at 4 months.	−0.02	−1.67	0.23
Penn DL et al., 2003 [55]	USA	163	18.85	College students (general major)	Controlled clinical trial	Documentary on Schizophrenia	70 min (once)	1 (immediately after)	No significant impact on intended behaviour. Less blame and responsibility for their disorder was placed on people with schizophrenia (p < 0.05).	–	−1.6	−2
Hecht ML et al., 1993 [43]	USA	465	14–18	School students	RCT	Film docudrama and live musical (Killing Time)	34 min (once)	1 (1 month post-test)	Discussion in addition to watching the film did not impact negative attitudes towards drug use, but increased confidence to resist drugs. Discussions neither detracted nor added to the effectiveness of film.	–	–	–
Hawke LD et al., 2014 [42]	Canada	28	21.2 (2.5)	College students (Health professionals in-training)	Cohort (one group pre-post design)	That’s Just Crazy Talk – DVD of a filmed play	50 min (once)	2 (immediately post and after 1 month)	Participants desired less social distance over time (*p* = .012) and significantly increased student willingness to interact with individuals with Bipolar Disorder. No significant change in stigma-related attitudes. Characteristics of this intervention were not suited to youth.	–	−0.07	0.25
Jerome lW., 1992 [46]	USA	184	14–18	School students	Cohort (one group pre-post design)	Film presentation	NR (Once)	2 (post-test at three weeks and after 18 months)	Participants showed an increase in knowledge about bulimia (maintained at 18 months).	–	–	–
Reis J et al., 2000 [59]	USA	4695	16–25	College students (general major)	Cohort analytic (two group pre and post)	Interactive software with animation, and three videos with choices	NR (Once)	NR details of repeat measures	Less positive attitude towards alcohol’s effects. Increased knowledge of symptoms of overdose, when to intervene, how many drinks it may take to reach intoxication (significant).	–	–	–
Kerby J et al., 2008 [51]	UK	46	21	College students (Health professionals in-training)	Controlled clinical trial	Two anti-stigma films to challenge stereotypes	27 min (once)	2 (immediately post and after 8 weeks)	Reduced social distance in the intervention group over the three time points (p < 0.001). Scores significantly increased at follow up (*p* = 0.03). Between baseline and post-test there was a significant decline in stigmatizing attitudes (*p* = 0.009).	–	0.75	−1
Tucker JB et al., 1999 [64]	USA	115	5–8 grade	School students	Post-test only for one group	Videos on violence, dealing with anger and gunshot victim	4 components, no info on duration	1 (immediately post)	Recall and identification of violence as a problem was high. 90% of students correctly identified the main message. Commercial and rap music video rated higher than trauma resuscitation video and discussion of anger.	–	–	–
Woods DW & Marcks BA, 2005 [68]	USA	180	22.33 (5.89)	College students (Health professionals in-training)	Controlled clinical trial	Video clips of a person with Tourette Syndrome and depression	20 min (once)	0	Higher social acceptability among the experimental group for people living with Tourette Syndrome.	–	–	–
Irving LM & Berel SR, 2001 [45]	USA	110	18–38	Female college students (general major)	RCT	Slim Hopes video	45 min (once)	1 (immediately post)	Participants were more sceptical about media images, related to body image.	–	− 0.8	–
Chan J et al., 2009 [33]	Hong Kong	255	14.6	School students	Controlled clinical trial	The Same or Not the Same- featuring life experience of four 18–24 year olds diagnosed with schizophrenia	NR (Once)	2 (immediately post and after 1 month)	Reduced social distance in the intervention group and more positive attitudes towards people with schizophrenia (*p* < 0.05). Participants in the education–video group had higher level of knowledge about schizophrenia than those in the video–education group (p < 0.05) at post-test. At follow-up, the effect size of the condition effect was moderate (p < 0.001).	−0.16	0.08	0.04
Fernandez A et al., 2016 [39]	Malaysia	102	20–23	College students (Health professionals in-training)	RCT	Video contact	40–45 min video & 1 h lecture (once)	1 (after 1 month)	Significantly reduced social distance and more positive attitudes between pre-test and post-test and after 1 month follow up (p < 0.001).	–	–	–
Conrad et al., 2014 [35]	Germany	515	15.6 [10–20]	Adolescent film festival	Cohort (one group pre-post design)	Five feature films and documentaries on mental health and wellbeing of adolescents	464 min (7.7 h)	1 (immediately post)	The effect on social distance and help-seeking attitudes towards people with mental health problems depended heavily on the respective film or documentary.	–	0.42	−0.01
Koike, et.al., 2018 [52]	Japan	259	20 (1.2)	Young adults in the general population	RCT	Repeated filmed social contact	30 min	6 (immediately post followed by every two months)	A sustained effect on reducing stigma, measured by a scale of intended behaviour towards people with mental illness.	–	–	0.7
Petkari, 2017 [83]	UAE	26	20 (1.4)	Psychology students	Mixed methods	Film followed by 1 h moderated discussion	10 weeks	1 (immediately post)	No significant differences in overall attitudes towards people with mental illness (^a^negative difference indicates lower stigma at post-test (see column. To the right); specific perceptions changed significantly.	–	−1.36^a^	− 1.15
Ta Park, et al., 2020 [85]	USA	118	22.1 (1.6)	College students	Mixed methods	16 episodes of School 2013, a Korean drama		1 (immediately post)	Knowledge, attitude and behavior towards bullying changed. Participants reported that they “love” the drama, felt an emotional connection, and realized that mental health issues are stigmatized topics. Participants want to see stress, depression and emotional strain addressed in the K-drama.	0.12	0.07	0.06
D. Studies using role play [3 studies]												
**Author/s, Year**	**Country**	**Sample size**	**Age [mean (SD)/range]**	**Participant profile**	**Study Design**	**Brief intervention description**	**Intervention Duration (frequency)**	**Number of follow-ups (times)**	**Description of change in all stigma dimensions (knowledge (K), attitude (A) and behaviour (B)**	**Change in stigma** *(Difference of means between experimental and control groups)*		
										K	A	B
Kimber B., 2012 [70]	Sweden	561	7–10 & 11–16	School students	Post-test only for one group compared to	Didactic sessions and role play	45 min for one year (weekly)	2 (after 2 years and after 5 years)	Medium effect sizes for a significantly more positive body image among 11–16-year-olds, compared to 7–10-year-olds.	–	−0.3	
King KA et al., 2011 [71]	USA	1030	14.1 (0.78)/ 14–18	School students	Cohort (one group pre-post design)	Role play and discussion	50 min (four sessions)	2 (immediately post and after 3 months)	Significant increase in likely behaviour to inform an adult of suicidal feelings of self or friends across all time points.	–	–	–
Roberts LM et al., 2008 [72]	UK	332	19–25^a^	College students (Health professionals in-training)	RCT	Role play	40 min (once)	1 (1 week post)	Significant increase in desired social distance, but no change in attitude towards people with mental health problems. Significant changes by gender (female) and people with previous experience of mental health problems.	–	−0.24	0.29
E. Studies using other art forms (dance/creative writing/music) [3 studies]												
**Author/s, Year**	**Country**	**Sample size**	**Age [mean (SD)/range]**	**Participant profile**	**Study Design**	**Brief intervention description**	**Intervention Duration (frequency)**	**Number of follow-ups (times)**	**Description of change in all stigma dimensions (knowledge (K), attitude (A) and behaviour (B)**	**Change in stigma** *(Difference of means between experimental and control groups)*		
										K	A	B
Salmon D et al., 2005 [76]	UK	249	14.3/ 11–19	School students	Qualitative research	Dance performance competition	One day (once)	2 (at the finale and 1 month after)	Recalled drug-free messages and pledge	–	–	–
Frey KS et al., 2005 [79]	USA	1023	7–11	School students	Mixed methods (on a controlled clinical trial)	Steps to Respect –(Creative word and literature lessons)	3 h (weekly)	2 (at 6 months follow up)	Bullying behaviour decreased. The experimental group found bullying and aggression less acceptable.	–	−0.11	−0.09
Harris, et al., 2019 [80]	USA	62	18–29/ 20.5	Undergraduate college students	Mixed methods (based on four cohorts)	Intergenerational choir rehearsals, socialization with people living with dementia and a concert	10 weeks (90 min rehearsals per week)	2 (half-way; post-test)	Use of more positive words to describe images of people living with dementia (55% change from negative words to positive) at post-test; improved understanding of dementia, avoiding labels, recognizing capabilities	–	–	–

^a^estimated based on educational level suggested in study

Nearly all studies were conducted in high-income countries, 44% were located in USA, 26% in the UK, and 9% each from Canada and the rest of Europe, and another 9% from Hong Kong, Japan, Australia and UAE. Only three studies were carried out in upper-middle-income countries of Brazil, [81] Turkey, [30] and Malaysia [39]. No studies were conducted in low-income countries. Six studies were published before 1995, 16 studies from 1996 to 2005 and 35 studies were published after 2006. Over half the studies focused on middle and high school students (53%), and the remaining studies targeted college students (42%) and youth in the community (5%). About 72% of college-based studies concentrated on health professionals’ in-training (medicine, health sciences, psychiatry, psychology, pharmacy or nursing) [30, 32, 34, 38–40, 42, 50, 51, 58, 68, 72, 77, 78, 81, 83]. Diverse stigma assessment measures were used by 33 of all quantitative and mixed methods studies (including modified instruments), [30, 31, 33–35, 37–40, 42–45, 47, 49–52, 54, 55, 57, 62, 63, 65–68, 71, 72, 79, 83–85] while 5 remaining studies used newly developed measures [36, 58, 61, 64, 69] and 13 studies did not specify instruments used or used informal/oral feedback or open-ended questions [32, 41, 46, 48, 53, 56, 59, 60, 70, 80–82, 86].

### Study designs

#### Quantitative studies

Seventy-five percent (*n* = 43) of included studies used a quantitative design. Eight studies were randomised controlled trials (RCT) [31, 34, 39, 43, 45, 52, 53, 72]. Other quantitative studies include 10 controlled clinical trials, [33, 38, 40, 49–51, 54, 55, 57, 68] nine studies used a two group, pre-post design, [30, 41, 47, 58, 59, 61, 66, 67, 69] 12 studies used a single group pre-post design, [35–37, 42, 44, 46, 56, 60, 62, 63, 65, 71] and four studies employed a post-test only design [32, 48, 64, 70].

#### Qualitative studies

Of six qualitative studies, one did not clearly define the method of qualitative research, [73] two used in-person and/or telephonic semi-structured interviews, [75, 77] a study used focus group discussions (FGD), [76] another used ethnographic procedures, [74] a study used students’ reflective essays, short films, and course evaluations, [78] and two used field notes and observation [74, 76].

#### Mixed methods studies

Mixed methods were used to supplement studies, which were overtly quantitative: a RCT, [86] a controlled clinical trial, [79] a one-group pre and post study [80, 83–85] and two qualitative studies, included surveys [81, 82]. These studies reported the use of observations [79, 82, 86] open-ended questionnaires, [79, 82–84, 86] semi-structured interviews, [80] group interviews, reflective groups and FGDs, [81, 82] drawing and explanatory writing, [84] and field notes [86].

### Intervention design

#### Quantitative studies

Eighteen quantitative studies involved multiple art forms (creative writing, role-play, theatre, film/ slideshow, collage), [30, 32, 36, 37, 40, 44, 48–50, 53, 54, 56, 58, 62, 63, 66, 67, 69] 15 studies involved film (including two RCTs), [31, 33–35, 39, 42, 43, 45, 46, 51, 52, 55, 59, 64, 68] eight used theatre (including puppetry and stand-up comedy), [38, 41, 47, 57, 60, 61, 65] and three used role-play [70–72]. Complementary lectures and educational material were used in 16 studies [30, 32, 33, 36, 39, 40, 44, 49, 50, 53, 58, 63, 66, 67, 69, 70] and social contact was included in eight studies [30, 31, 33, 34, 39, 42, 51, 68]. Professionally created art was used in 34 (79%) of studies [30–38, 40–48, 50–52, 54–56, 58–62, 64, 66–68, 72] and only nine studies reported some form of voluntary participation or participant input [34, 38, 45, 47, 51, 56, 57, 65]. Twenty-four studies evaluated single sessions (mostly of 1 hour’s duration) [30, 32–34, 38, 39, 41–48, 50–52, 55–57, 61, 63, 68, 72] and 12 studies reported between two six sessions, [31, 36, 41, 49, 54, 58, 60, 62, 65–67, 71] and in the remaining seven interventions, the number of overall sessions is not clear [35, 37, 40, 59, 64, 69, 70].

Among eight RCTs, varied combinations of stigma components improved in a majority of studies, except a study using role play which reported no significant change in any aspect of stigma [72]. Only one RCT measured and improved all components of mental health stigma (KAB) using film [34]. Other RCTs improved: attitude and behaviour using film [39]; knowledge and attitude using multiple art forms [53] and film [31]; attitude using film [43, 45]; and behavior using film [41, 52, 56]. Intervention content included facts on incidence, causes, symptoms and warning signs of mental illness, broadly and about specific disorders, including bipolar disorder, schizophrenia, depression, substance abuse and suicidal behaviours. Social contact was used to describe negative experiences of stigmatisation using filmed or dramatised interventions. Even single session interventions included multiple art forms [30, 48, 63].

#### Qualitative studies

From the six qualitative studies, three studies employed theatre interventions, [74, 75, 77] two used multiple arts forms, [73, 78] such as music, radio, documentary and visual arts or students’ reflective essays and short films [78] and another used dance [76]. In four out of six qualitative studies, participatory or collaborative approaches involved students as performers of art or as collaborators in co-creating art with persons living with mental health problems [73, 75, 76, 78]. Only one intervention study comprised a single session [74] and other the other five studies involved multiple sessions, with intervention duration ranging from 2 weeks [73] to 8 months [75].

The study reporting a positive improvement in all components of mental health stigma (KAB) used a professional play, followed by role-play [74]. Among other studies, two using theatre, [75, 77] one using music and visual arts [73] and one using dance [76] suggest positive gains in knowledge and attitude (KA) related to mental health problems and drug use, and reducing awkwardness and increasing empathy. The study involving music and creation of short films on dementia reported that 27% of participants continued to volunteer in dementia care settings after the intervention was completed [78].

#### Mixed methods studies

Six out of eight studies involved professionally created art, [79, 81, 83–86] while the other studies helped youth create their own rap songs [82] and participate in a choir [80]. Three interventions used multiple art forms (film, theatre, rap songs, role play and educational materials) [81, 82, 86] and one each used children’s books, [79] song lyrics, [81] film [83] and interactive theatre [84]. Collaborative art or co-created art was evaluated using a post-only survey and interviews [82]. Only one study used an intervention that lasted a single day [84] and other interventions ranged from between 4 weeks [86] to 10 weeks [80, 83]. In another study follow-up material was mailed to participants for 12 months after the intervention [85]. Only one mixed-methods study stated that youth participation was voluntary [82].

Three studies report changes in all components of mental health stigma (KAB), one using theatre, [84] another using film [85] and another other using multiple art forms [86]. These studies include knowledge about drugs, mental health awareness and self-recognition; attitudinal change that anyone could be affected by mental health problems and behaviours such as reduced negative words and desire to help those in need (intended behaviour). A study each improved acceptance and bystander responses (AB), [79] and knowledge about substances (K) [81]. and two studies improved knowledge and attitudes related to substances and dementia (KA) [82, 80].

### Risk of bias

#### Quantitative studies

**Fig. 2:**
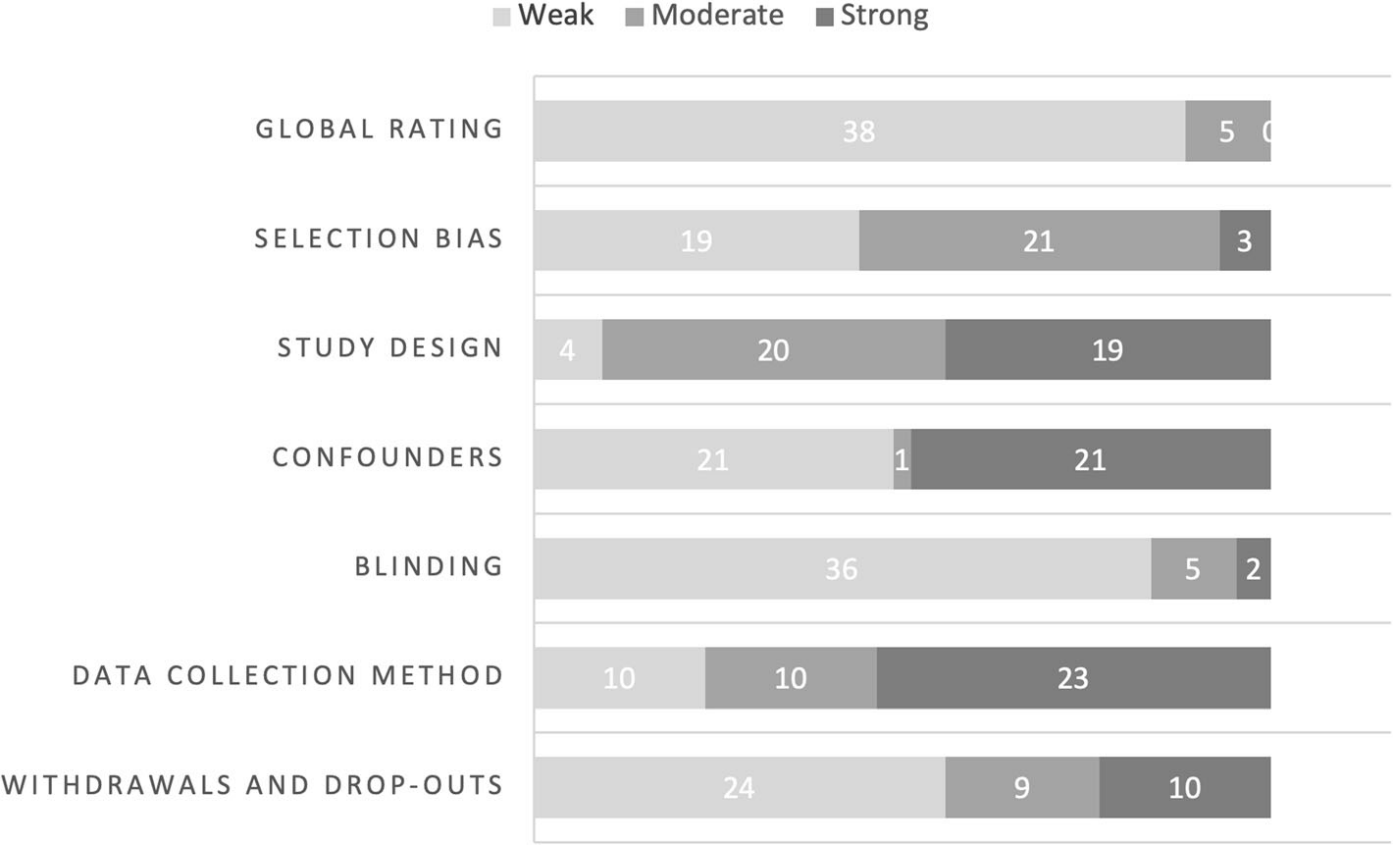
Study quality of quantitative studies (risk of bias as per EPHPP tool) (*n* = 43)

Overall, study quality rated using the EPHPP tool [26] ranged from weak to moderate, with some studies displaying strong methodological aspects (Fig. 2). A detailed quality rating of included studies is in supplementary Table 3. Regarding study design, eight studies were accurately described as randomised controlled trials (RCT), [31, 34, 39, 43, 45, 52, 53, 72] 20 were quasi-experimental studies with control groups, while the remaining had weaker designs. Participants were not representative of the population in 17 studies, mostly because they self-selected [35–38, 41, 42, 44, 45, 55–57, 60, 62, 65, 67, 69] and were partially representative of the population in another 17 studies, where participants were referred from a school or university [30, 31, 33, 40, 43, 46, 47, 49–51, 54, 58, 59, 61, 63, 66, 71]. Six studies had participation rates greater than 80%, [35, 58, 61, 63, 72] six studies had participation rates between 60 and 79%, [30, 33, 34, 46, 47, 54] 11 studies had participation below 59%, [38, 45, 50–52, 57] and remaining studies did not report participation rates. Studies had varied drop-out rates, the highest being 59% [60].

Researchers were blinded to participant exposure in four studies [39, 50–52] and in 16 studies participants were unaware of study aims [33, 34, 39–42, 45, 47, 50, 51, 54, 55, 58, 61, 68, 71]. Only 17 studies reported and adjusted for potential confounders [30, 33, 38, 40, 41, 44, 45, 47, 50, 52, 54, 55, 60, 61, 63, 67, 69]. Seven studies used data collection instruments that were not validated, [35, 41, 46, 56, 59, 65, 69] of which two studies established reliability of instruments used [35, 59]. Of the remaining 36 studies using validated instruments, 10 did not establish reliability [30, 37, 43, 44, 49, 57, 60, 63, 66, 70]. Approximately half of quantitative studies (53%) did not follow up after post-test (typically 1 month or immediately post-test) (*n* = 43) [30, 33, 35, 37, 38, 41, 43–45, 49, 50, 55–58, 61, 63, 65–69, 72]. Several studies in this review highlight short-term measurement of impact as a limitation [31, 35, 37, 39, 41,51, 57, 58, 64, 67, 79, 86].

Overall, studies using film had good quality, studies using theatre had moderate quality and studies using multiple art forms and role play had weak study designs. Confounders were better addressed by studies using theatre and multiple art forms, compared to role play and film. Valid and reliable data collection instruments were used by studies using theatre and film, followed by multiple art forms and lastly, role play.

#### Qualitative studies

From six qualitative studies, a study lacked quotations to assess validity of conclusions, [73] and one study presented quotations as a response to questionnaires [74] [87]. One study reported full participation, [75] rate of participation varied from 10 to 88% in three studies [74, 76] and was not specified in the remaining three studies [73, 77, 78].

#### Mixed methods studies

Out of eight mixed methods studies, one included a cluster randomized controlled trial, [86] seven reported quantitative outcomes, [79–85] however only two studies included sufficient qualitative data [82, 84]. Participant response rate was not specified in five studies, [81, 82, 84] [80, 86] below 60% in one study [83] and above 80% in three studies [79, 85].

### Synthesis of results

#### Outcome measures

**Fig. 3:**
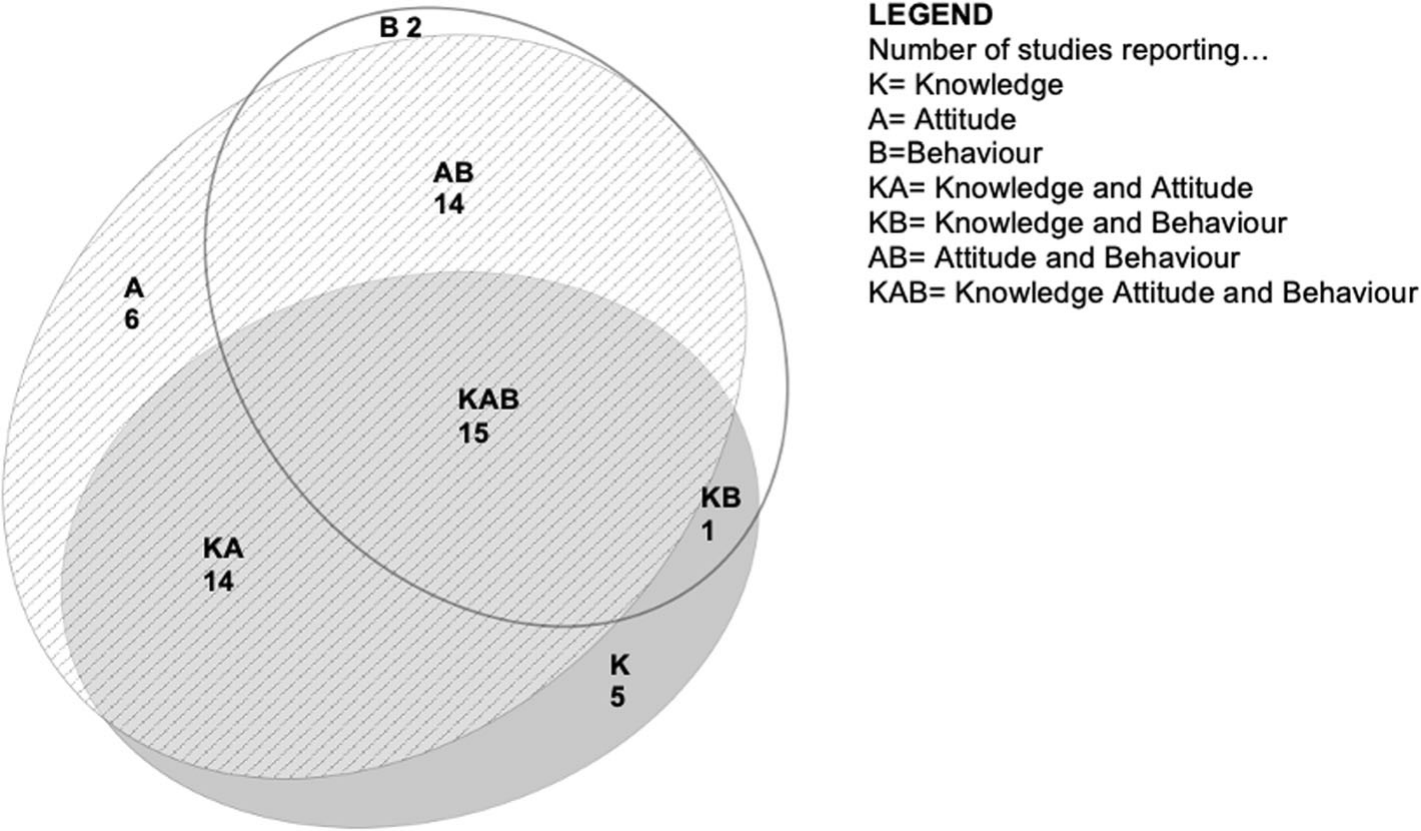
All studies, by combinations of stigma outcomes reported (*n* = 57)

All 57 studies reported various combinations of mental-health-related public stigma components as outcomes, i.e., knowledge, attitude and intended behaviours (see area-proportional Venn diagramme [88] in Fig. 3). Six out of eight studies with a randomized controlled study design reported a significant positive change all stigma components reported, [34, 39, 43, 45, 52, 53] including one RCT which reported positive, significant effects on all knowledge, attitude and behaviour outcomes (KAB), [34] another RCT on attitudes and behaviors (AB), [39] one RCT on knowledge and attitudes (KA), [53] two RCTs on attitudes [43, 45] and one on behavior [52]. Of the remaining two studies, one found no significant difference in AB [72] and another reported no change in behaviour in a study reporting all KAB components [31]. In 10 controlled clinical trials of strong study design, four studies reported positive significant effects on KAB, [33, 40, 49, 50] three studies reported positive significant effects on AB, [38, 51, 68] two reported positive significant effects on attitudes [54, 57] and only one reported no significant effect [55]. Per the EPHPP risk of bias assessment, two studies of moderate global study quality show positive effects on AB, [39, 68] two show positive effects on attitudes [54, 57] and one showed a sustained effect in reducing stigmatizing behavioral intent [52]. Seven studies collected follow-up data an average of 4 months post-intervention [33, 34, 38, 43, 51, 54, 66]. Two of these seven studies showed positive and significant results on KAB at follow up, [33, 34] and the remaining studies on attitude and behaviour [38, 43, 51, 54, 66]. Nearly all quantitative studies (*n* = 40 out of 43) reported positive changes on at least one stigma-related outcome, including 12 studies with strong study design quality [33, 34, 38–40, 43, 45, 49, 51, 54, 57, 68] and 17 studies with moderate study quality [30, 36, 37, 41, 42, 47, 58, 60–63, 65–67, 69, 71]. No study reported a negative outcome.

Out of six qualitative studies one reported positive changes in KAB, [74] three studies improved knowledge and attitudes (KA) associated within mental health [75, 77, 78] and the remaining two studies improved knowledge (K) by way of recall and the level of awareness about mental health problems [73, 76]. Three studies focused on reducing stigma associated with drug-related issues, [73, 74, 76], one study on dementia, [78] one study on depression, anxiety, panic and stress, [75] and one on trauma [77]. Studies focused on the process of using art as a stimulus for discussion and narratives focused on achieving attitudinal change, positive self-esteem, purpose and satisfaction from participation***.*** Some of these studies highlight the emotional impact of art as a tool to relate stories and personal experiences, [77, 78] changes in how youth use labels and describe interactions with people living with mental health problems, [75] and one reportedly led to substantial increases in requests for counselling [74].

From five mixed methods studies, three studies reported positive results on KAB, one study reported positive impact on attitude and behaviour (AB), [79] another two studies reported positive changes in knowledge and attitude (KA), [82, 86] one study improved specific attitudes only [83] and lastly, one study improved knowledge [81].

### Meta-analysis

#### Effectiveness of art in reducing components of stigma

**Fig. 4:**
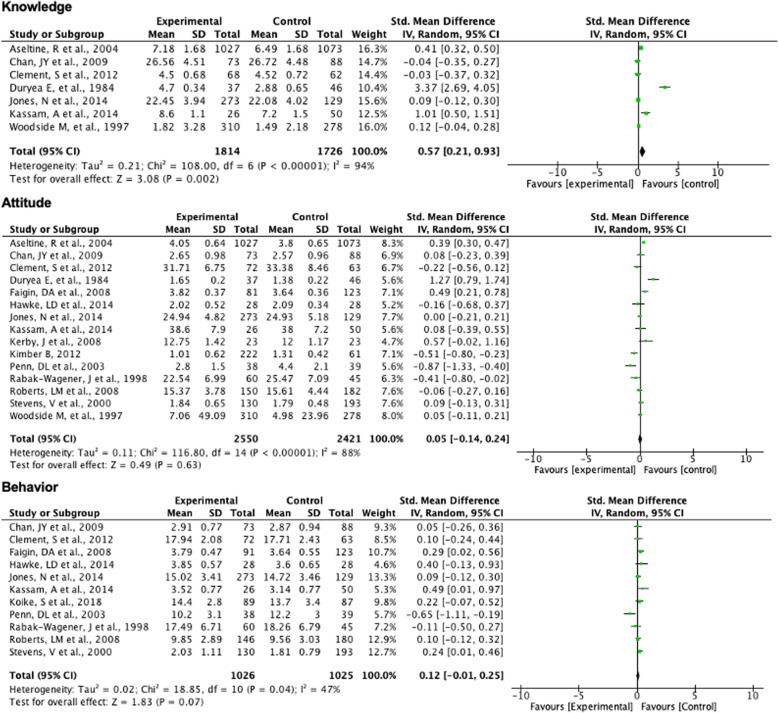
Meta-analysis of arts interventions on mental-health-related knowledge, attitude and behaviour

There was no significant difference in whether arts interventions improved behaviour towards people with mental health problems compared to a control group (effect size = 0.12, 95%CI -0.01-0.25; *p* = 0.07) (Fig. 4), and moderate heterogeneity was reported across studies (I^2^ = 47%). High heterogeneity of studies on knowledge and attitude outcomes made meta-analysis inappropriate (88–94%).

#### Effectiveness of different art forms

**Fig. 5:**
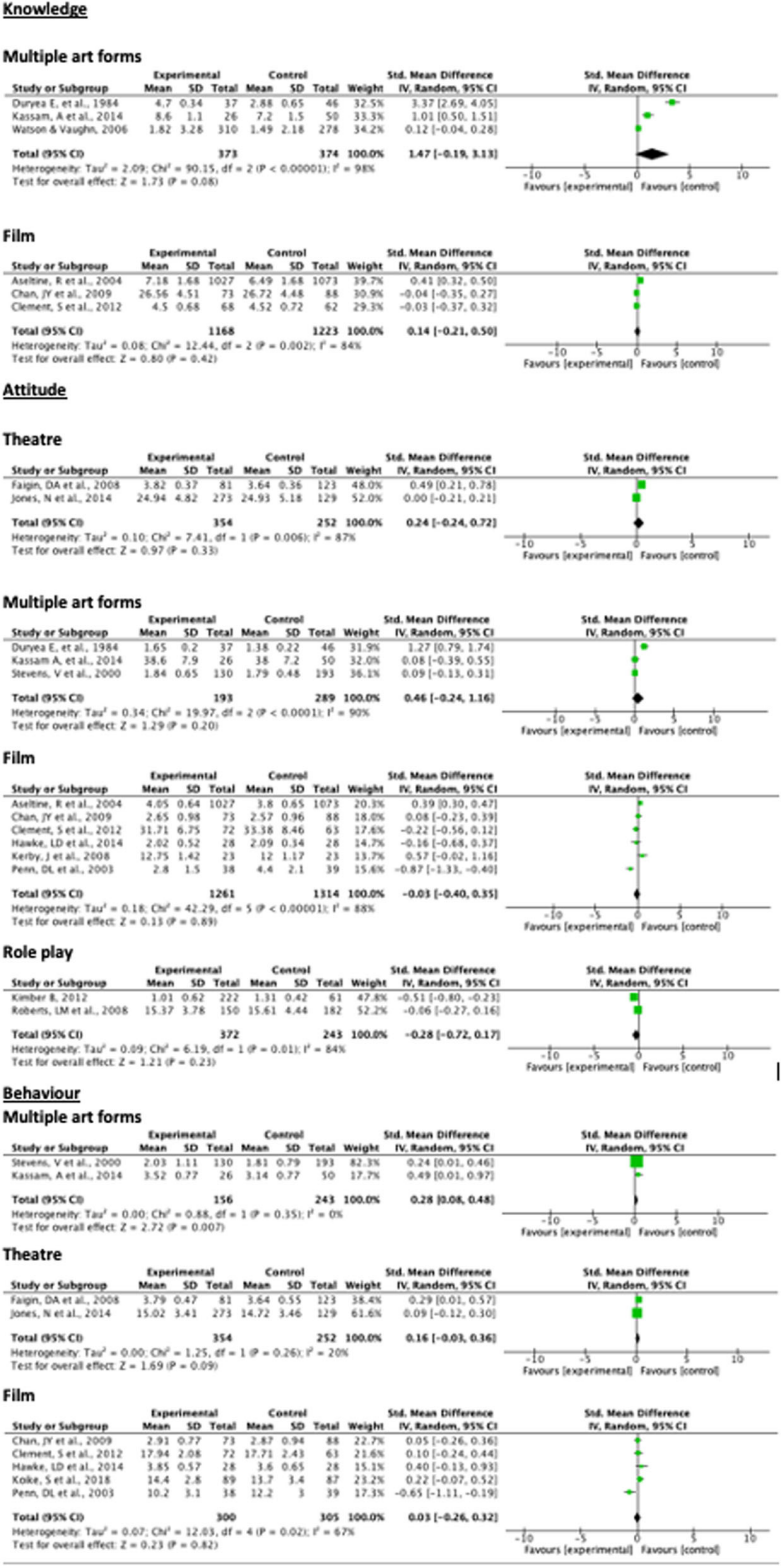
Meta-analysis of mental-health-related knowledge, attitude and behaviour, by intervention art form

The largest positive effect on knowledge may be attributed to interventions using multiple art forms (effect size = 1.47, 95%CI -0.19-3.13; *p* = 0.08), followed by film (effect size = 0.14, 95%CI -0.21-0.50; *p* = 0.42) (Fig. 5). However, the I^2^ value for pooled studies in this meta-analysis, reporting knowledge-related outcomes, was between 84 and 98%. No data were available for theatre, role play or other studies with respect to knowledge. Similarly, the impact of interventions using theatre, film, multiple art forms and role play on changing attitudes was not significant. Studies pooled by each of these art forms had heterogeneity, ranging from I^2^ = 80–94%. Interventions using multiple art forms were the only ones that significantly reduced stigmatising, practised or intended behaviours (effect size = 0.28, 95%CI 0.08–0.48; *p* = 0.007) (Fig. 5). Theatre-based interventions pooled by behavioural outcomes showed low heterogeneity (I^2^ = 20%) and film-based studies pooled by behavioural outcomes showed moderately high heterogeneity (I^2^ = 67%). No data were available from studies using role play and other art forms, due to lack of precise measurement or poor quality of reporting.

#### Effectiveness of interventions by duration

**Fig. 6:**
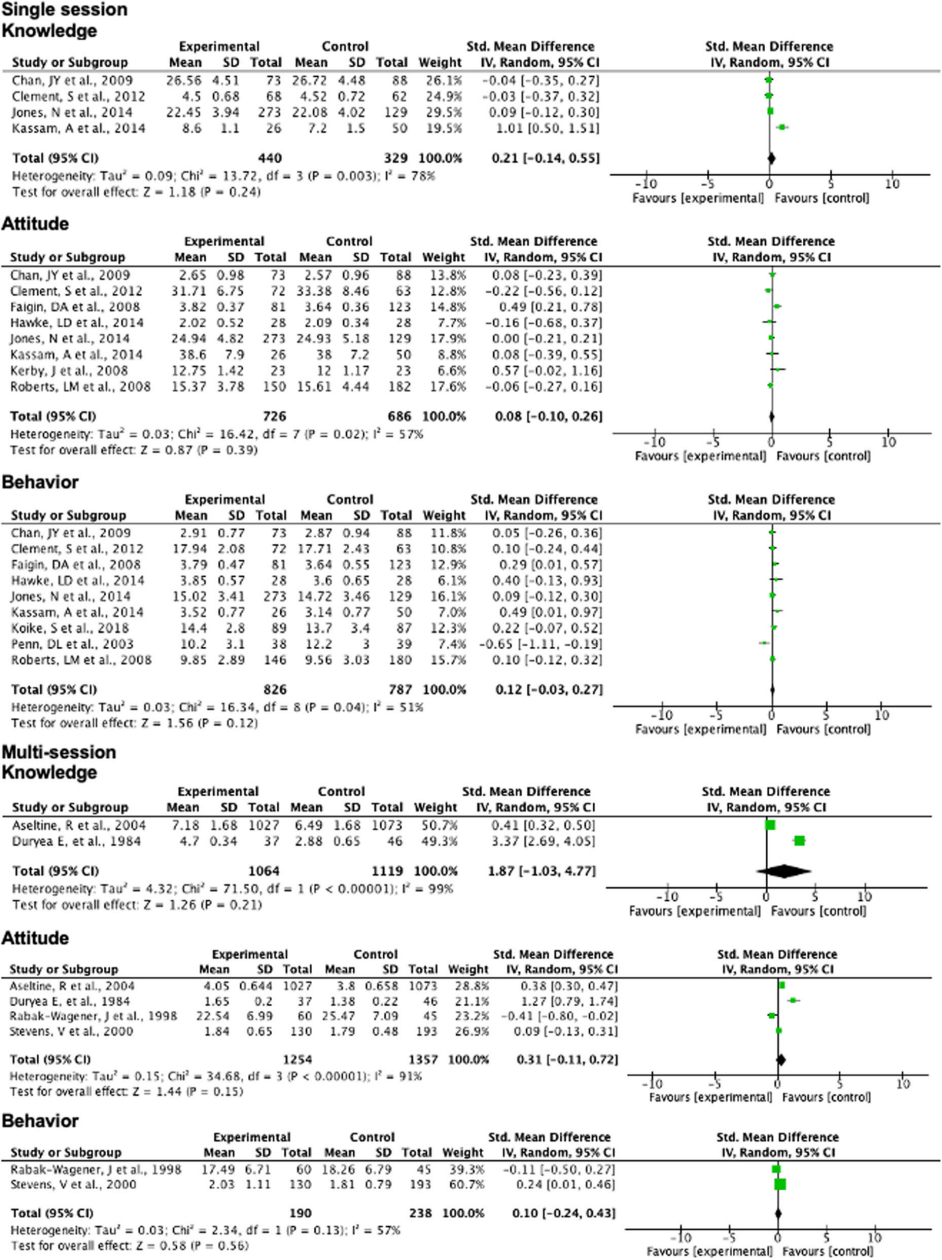
Meta-analysis by intervention duration and studies focusing on all stigma components

Studies pooled by duration, i.e., whether single session interventions or multi-session interventions, displayed moderate to high heterogeneity (I^2^ = 51–99%) and did not show any significant effect on knowledge, attitude or behavior (Fig. 6).

#### Comprehensive approach versus focus on individual stigma domains

Studies that took a comprehensive view of stigma showed no significant improvement in behavior (effect size = 0.12, 95%CI -0.03-0.27; *p* = 0.11). The I^2^ = 0% indicates that pooled studies had low heterogeneity, and therefore that the meta-analytic approach was appropriate (see Supplementary material 4: figure). These studies focused on all components of knowledge, attitude and behaviour (KAB) in measurement and possibly also in intervention content. For knowledge- and attitude-related outcomes in studies reporting KAB, there appeared to be a positive effect (effect size = 0.09–0.25), but there was high heterogeneity among pooled studies (I^2^ = 84%) and these results were not significant. There were no studies that focused on knowledge, attitude and behavior components of stigma alone and that met study design quality benchmarks for meta-analyses.

#### Barriers and facilitators in implementation and reducing stigma

Overall, multiple mechanisms and contingencies were reported to influence implementation and participant engagement, especially attendance and quality of delivery. Fluctuating intervention attendance, [82] awkwardness and scepticism, [57, 59, 86] language-related issues, [74, 82] group and gatekeeper dynamics [57, 66, 75] and logistical issues [70] influenced implementation in several studies. In a large number of studies females were overrepresented [32, 38, 42, 45, 50, 51, 67, 75, 76]. Unintended consequences were reported in a study using multiple art forms (professional theatre, quiz and games) with school children, where mental health problems were normalised to the extent that participants felt that these problems did not have much of an adverse impact [37].

Active ingredients that facilitate successful delivery of arts-based interventions include institutional endorsement for the initiative at educational institutions [39, 75] and scheduling sessions during class times [31, 38, 41, 49]. Clear content, [55, 68] a diversity of views from presenters [34, 66] and involving people with mental health problems for embedded social contact were perceived to reduce stigma [31, 34, 42, 50]. Visual stimuli and expressive arts-based techniques were useful tools to facilitate participation [64, 86]. Further, high quality, emotionally powerful art performed may help achieve a stronger, anti-stigma stance among participants. Thus, several studies highlight the value of involving professional artists [37, 60, 73, 82, 84]. In one study that involved youth in performing scripted theatre, [54] the authors observed that even deeper participation was needed to reinforce key ideas. A study which enabled youth to act in scripted plays highlighted the importance of public reinforcement of messages through performance, however to align content to participant experiences, it recommended that participants write their own scripts [54].

Youth arts projects meet social needs of young people to engage in a popular programme [76]. They involve people external to educational institutions, which studies felt youth appreciate [65]. Additionally, youth projects accord an equal status among participants, [39] which according to Fernandez et al. is ideal for ‘cooperational education,’ where students learn and evaluate key programme messages collaboratively. Such interventions simultaneously use skills-building and educational appeals, rather than purely emotional ones, an approach which has been suggested as longer lasting. The Studio 3 Arts project among 13–21 year olds in the United Kingdom created participatory music, radio, documentary and visual arts for drugs-related awareness [73]. However, findings were inadequately reported from the perspective of effective intervention components. The project was reported in a brief, non-technical, magazine style which described the process and provided limited participant quotations or summaries of their experience as support. A pilot mixed methods study of VoxBox, co-creating rap music with high school students in Australia showed non-significant positive changes in knowledge, attitude and intended behaviour related to alcohol users [82]. Twardzicki et al. conducted a study in the UK in 2008 which generated theatre productions through discussion between people with mental health problems and college students [65]. Rowe et al. conducted a similar study in 2013 with students belonging to a theatre major, who co-created art on the theme of mental health with users of mental health services [75]. Although this study had a small sample size, its authors suggest that ‘shared, theatre-making may create an environment that challenges stereotypes and reduces prejudice.’ [75] Studies that used participatory, co-creation of art in this review, predominantly used qualitative and mixed methods for evaluation. These studies also demonstrate the impact of youth participatory arts projects focused on mental-health-related public stigma as a theme on critical thinking, problem-solving and building team spirit.

## Discussion

### Summary of evidence

This review finds positive indications for the use of art to address mental health stigma among youth. Although, strong assertions about effectiveness are not plausible given poor methodological quality of studies, results from this meta-analysis are indicative of a direction of travel supporting the effectiveness of art-based interventions.

Arts interventions are generally effective when they use multiple art forms, but with a small effect. This study also demonstrates that we do not affirmatively know whether interventions with multiple sessions had a greater effect on stigma, relative to single day interventions. Further, it remains inconclusive whether a comprehensive approach to stigma (including all stigma components of knowledge, attitude and behaviour in an intervention study), translates to significant improvements in knowledge, attitude and/or behaviour relative to studies focused on changing each of these individual stigma components. Common challenges faced by interventions related to buy-in from school or college stakeholders and youth engagement. No studies were reported from low- and lower-middle-income countries, and this highlights the need to develop, and report results from arts-based interventions in those contexts. No studies reported negative outcomes or unintended harms.

This review does not provide evidence to support conclusions from reviews by Schachter [13] and Mellor [12] on school-based interventions to reduce mental health stigma, that use of multiple art forms may coincide with multiple exposures and a more intensive engagement. The use of multiple art forms may have attracted and engaged participants with varied interests to reinforce concepts related to the theme of mental-health-related stigma. Overall, the most commonly reported underlying theory is Bandura’s social learning theory, where youth are likely to emulate [89] less stigmatizing behaviour if they observe stereotypes or are able to concretize their experiences through art. Film-based studies were too heterogenous, likely due to varying educational content including filmed theatre or social contact or documentary; varying duration of films and varying complementary activities such as discussion or role-play.

Multiple art forms are potentially more impactful than other art forms in lowering stigma as a combination of art forms likely aims for a more intense experiences compared to use of a single art form [90]. These programs have the potential for greater interactivity and longer duration as well as the possibility of attracting youth who may be interested in using or engaging with at least one art form among several deployed. Findings related to the effect of theatre and role play in this review, are supported by Joronen’s review on school-based drama, which showed short-term effects on health-related knowledge and behaviour [91]. Our findings related to implementation barriers such as inconsistent participant attendance may be overcome by recommendations by authors of included studies to use participatory student arts-based projects that involve direct youth engagement. Given that including voluntary role play as 20% of an intervention on mental illness led to changes in youth knowledge and attitudes in a recent study, [92] one may expect a positive response and increased acceptability in studies where participant-created art is a complementary component. However, most intervention studies in this review involved mandatory attendance of professionally created art.

In this review, collaborative art or co-created art involving students was evaluated using mixed methods (post-test only for one group) or qualitative research. Other recent studies place the responsibility of creating art directly in the hands of young people through a variety of art forms: photo-voice; scripting, filming, and editing a public service announcement targeted to peers, and words and messages in response to a participatory public art project on mental health [93–96]. Study outcomes relate to enabling participants to describe their perceptions in relation to mental health, share personal experiences of stigmatized topics and the ability to participate in a project that validates that mental illness is real and acknowledges the need for shame-free mental health awareness [95]. As more rigorous evaluations of these participatory interventions are conducted, and an expanded range of outcomes are studied, their effectiveness in changing participants’ knowledge, attitudes and behavior associated with people with mental health problems will become clearer.

Most arts-based interventions target health professionals in-training. College students from other backgrounds should justifiably have access to age-appropriate interventions on mental health stigma for prevention, early detection and acceptance of people with mental health problems. Further, three studies in this review observed that their interventions were likely more effective for older adolescents compare to children, [62, 69, 79] potentially because older adolescents have the confidence to communicate and skills to analyse complex, social and individual emotional responses.

### Study strengths

This review is unique because it collates evidence on pragmatic dilemmas of mental health promotion faced by policy-makers, researchers, practitioners and communicators/educators. It is also unique in its comprehensiveness, as it explores the effectiveness of arts-based interventions across a range of mental health stigma-related outcomes, study designs, art forms and intervention durations. This review takes a broad view of art and mental-health-related stigma. Other systematic reviews of interventions in mental health prevention include creative, artistic or entertainment techniques, and also do not acknowledge them as ‘art.’ [97–113] Many studies in this review use arts-based interventions, but do not explicitly recognise or state that they use art, expand on the purpose of art or define a clear pathway to change or theory of change through arts interventions. This review included all such studies in addition to including a wide range of arts-based techniques and mental health conditions.

This review examines the theoretical understanding that comprehensively addressing all components of stigma is likely to impact intended behaviour towards people with mental health problems and towards help-seeking, rather than focusing on knowledge or attitude alone. The sub-group analysis by stigma components, extends the approach in a systematic review by Hanisch et al. in 2016, where they assessed and plotted successful impact on knowledge, attitude and behaviour outcomes from workplace interventions [114]. While duration of follow-up has been a subject of investigation for many systematic reviews, the impact of intervention duration (single vs multiple sessions) is explored by this review, although we observed inconclusive results.

### Limitations

As per the EPHPP tool, [26] none of the quantitative studies received a strong overall rating. Studies scored poorly in terms of blinding of researcher awareness to intervention allocation, and selection bias due to convenience sampling and participant self-selection, which is common in researcher-led communication or public engagement interventions. Our search returned few randomized and/or controlled trials assessing the effectiveness of arts-based interventions on mental-health-related stigma overall, and its components of knowledge, attitude and behavior. Since interventions are continuously being designed and developed, this review sought to analyse all available evidence to inform stigma-reduction initiatives amongst young people. Thus, we have included all studies (including quasi-experimental studies) of generally high quality in our meta-analyses, to identify a direction of impact, no impact or negative impact rather than focus on estimates of expected change in outcomes. Readers are encouraged to review confidence intervals and heterogeneity to gauge the level of certainty of expected outcomes when implementing a study using arts interventions.

Specific subgroup analyses were affected by high heterogeneity (I^2^ values). In addition, several studies provided inadequate data and therefore, were not pooled. For the sub-group analysis by duration, varying time-points for follow-up and lack of follow-up implied that studies could not be pooled and that only short-term effects at post-test (up to 1 month) could be feasibly calculated. Sub-group analysis by middle school, high school and university was not conducted due to fewer pooled studies. Other aspects that may have led to general heterogeneity include complementary components such as social contact [10, 13, 115, 116] and differences in measuring stigma. Finally, the concept of art, relationship of participants with observing and creating different art forms and therefore the relative effectiveness of interventions based on arts, are likely influenced by the cultural context in which such art interventions are applied. It was not feasible for this study to factor in cultural differences in how the impact of arts interventions vary across cultures.

Studies measured different combinations of mental-health-related stigma components. The most common methodological issue cited by nearly all studies was the extent to which participant responses were affected by social desirability. Several studies used intended behaviour as a reasonable measure of actual behaviour, since measuring actual stigma-related behaviour is challenging [31, 33, 34, 36, 47, 55, 79]. A study argued that intended behaviour consisted of beliefs, self-efficacy to act on those beliefs and perceived benefit from behaviour [62]. To address these issues, this review focused on a multi-pronged concept of stigma, which is more comprehensive (included a combination of knowledge, attitude and behaviour components) and also focused on intended behavior. If studies found that both knowledge and attitude or any combination of knowledge, attitude and behavior (as mental-health-stigma-related components) changed after an arts intervention, we found that such studies did not correlate or discuss the relationship between knowledge, attitude and behavior components. We believe these findings could be important for readers interested in implementing arts interventions who may need to understand whether incremental changes in knowledge may or may not be correlated with changes in attitude and intended behavior.

## Conclusion and implications

Overall, the studies reviewed demonstrate that arts interventions have limited effects on reducing young people’s discriminatory behaviour towards people living with mental health problems. The review specifically indicates that using multiple art forms in arts-based interventions likely impact youth behaviour towards people living with mental health problems. While the quality of evidence included in this review is modest, the number of interventions using arts-based methodologies and a strong direction of travel for impact on stigma indicate the scope for application of its findings.

This review identifies several opportunities to develop arts-based education to reduce mental-health-related stigma. First, the dearth of such interventions in low- and lower-middle- income countries calls for the development of new, contextual initiatives. Second, since most interventions are implemented in partnership with the education sector, school and college authorities should be sensitized to the need for mental health promotion and should consider including arts-based educational interventions as part of their curriculum. Third, interventions may focus on young adults in college and not just those who are training for healthcare-related careers. Fourth, student-led arts projects may be useful to explore mental-health-related stigma in an interactive format, which may then serve to reinforce social norms that are anti-stigma. Future intervention development may involve empirical development of student arts projects or participatory arts-based interventions to reduce stigma. Finally, robust, real-world evaluations are needed in the future that go beyond short-term follow-up periods.

The review suggests that conceptualization of art and content also require closer attention. For instance, the purpose of using art may be expanded beyond information-sharing to a transformative process, providing a sense of agency to participants to take supportive decisions and actions when confronted by a person with a mental health problem or attending situation. Student art projects or co-creation of art to reduce mental-health-related stigma may embody such a concept, and finds support in two theories: 1) Fisher’s communication narrative theory where art is a form of communication and storytelling and storytelling has the potential to re-shape the social world [117] and 2) Goldblatt’s interpretation of Dewey’s theory of art as experience, which highlights the transformative role of art in removing fear and prejudice, spurring critical analysis and empowering youth to achieve social justice [18]. With regard to content, future research on stigma-related theories may define conceptual boundaries between stigma components of knowledge, attitude and behaviour, and interrelations and possible hierarchies among these components. Such research would strengthen and guide intervention content, for example, by informing intervention planners whether a gain in knowledge about causes of mental health problems or change in attitude that people with mental health problems are to be feared could be instrumental in reducing negative behaviours, such as the use of harsh words against people with mental health problems. Such research must be based on cultural understanding and interpretations of mental health problems.

Notably, this is the first global review of arts-based interventions to reduce stigma associated with mental health problems. Practical and action-oriented findings from the review may inform anti-stigma interventions and other mental health promotion interventions using youth engagement strategies. Continuous knowledge-sharing of active ingredients in effective interventions and implementation research is needed to ensure the successful adaptation of arts-based interventions across settings.

## Supplementary Information

**Additional file 1.** Search strategy for arts-based interventions to reduce mental-health-related public stigma among youth.

**Additional file 2.** PRISMA checklist.

**Additional file 3: Table S3.** Quality rating of all quantitative studies using the Quality Assessment Tool from the Effective Public Health Practice Project (EPHPP).

**Additional file 4. **Meta-analyses of studies focusing on all KAB aspects of stigma (a comprehensive approach to measurement and possibly intervention.**content).**

## Data Availability

The data supporting the conclusions of this article are included within the article tables and figures.

## Abbreviations

PRISMA: Preferred Reporting Items for Systematic reviews and Meta-analyses
SE: Standard Error
CI: Confidence Interval
K: Knowledge
A: Attitude
B: Behaviour
