# Supplementary material for: Effectiveness of arts interventions to reduce mental-health-related stigma among youth: a systematic review and meta-analysis
Source: BMC Psychiatry. 2021 Jul 22;21:364. doi: 10.1186/s12888-021-03350-8 (PMC8296649; doi:10.1186/s12888-021-03350-8)
Supplement: Supplementary file 1 — Additional file 1. Search strategy for arts-based interventions to reduce mental-health-related public stigma among youth. [file 12888_2021_3350_MOESM1_ESM.docx]

**Supplementary Table 1. Search strategy for arts-based interventions to reduce mental health stigma among youth**

| **Category** | **Search terms** |
| --- | --- |
| Art | creativ* or express* or artistic or performing or performance arts or circus arts or theatre or drama or sketch or skit or dance or puppetry or mime or miming or magic or opera or improvisational theatre or pantomime or kabuki or ballet or stand-up comedy or visual arts or stage art or graphic arts or painting or choreography or sculpture or participatory media or "peer education" or roleplay or role-play or "role play" or community media or entertainment-education or entertainment education or songs or music |
| AND | |
| Mental health | mental or psychiatr* or anxiety or panic or bipolar or "personality disorder " or depression or dissociative or alcohol* or dependency or schizophreni* or mania or hypomania or learning disability or mood* or loneliness or obsessive compulsive or "self harm" or "self-harm" or paranoi* or phobia* or trauma or "post traumatic stress" or "self esteem" or "self-esteem" or sleep or insomnia* or stress* or suicid* or addicti* or bereave* or attention deficit or body dysmorphic or fatigue or delirium or delusion* or hallucination* or hyperactiv* or violen* or delinquen* or aggress* or bully* or victimi* or substance adj2 (use* or abuse or misuse) |
| AND | |
| Stigma | stigma or knowledge or awareness or myth or stereotyp* or attitude or prejudice or negativity or discriminat* or exclusion or distance or intended behavior or likely behavior or avoidance or communication or dialogue or interaction or confidence or emotional wellbeing |
| AND |  |
| Youth | youth or young or teen* or student* or university or college or undergraduate* or adolescen* or youth led |

*Symbol of truncation in order to search keywords with varying endings and plural forms.
