# Supplementary material for: Effectiveness of arts interventions to reduce mental-health-related stigma among youth: a systematic review and meta-analysis
Source: BMC Psychiatry. 2021 Jul 22;21:364. doi: 10.1186/s12888-021-03350-8 (PMC8296649; doi:10.1186/s12888-021-03350-8)
Supplement: Supplementary file 3 — Additional file 3: Table S3. Quality rating of all quantitative studies using the Quality Assessment Tool from the Effective Public Health Practice Project (EPHPP). [file 12888_2021_3350_MOESM3_ESM.docx]

Supplementary Table 3. Risk of bias assessment for quantitative studies using art [n=43]

| **Author(s)** | **Study Design** | **Rating for study quality** | **Rating for selection bias** | **Rating for confounders** | **Rating of studies on blinding** | **Rating of studies on data collection** | **Rating of studies on withdrawals and drop-outs** | **Global rating** |
| --- | --- | --- | --- | --- | --- | --- | --- | --- |
| Faigin DA et al | 2 | 1 | 3 | 1 | 3 | 1 | 1 | 3 |
| Aseltine, R.H et al | 1 | 1 | 2 | 3 | 3 | 1 | 1 | 3 |
| Chan, HV & Pervanas, HC | 7 | 3 | 3 | 3 | 3 | 3 | 3 | 3 |
| Clement, S et al. | 1 | 1 | 2 | 3 | 3 | 1 | 2 | 3 |
| Penn, DL et al. | 2 | 1 | 3 | 1 | 3 | 1 | 3 | 3 |
| HechtML, et al. | 1 | 1 | 2 | 3 | 3 | 2 | 3 | 3 |
| Duryea, E et al. | 5 | 2 | 3 | 3 | 3 | 1 | 3 | 3 |
| Gliksman, L et al. | 3 | 2 | 3 | 1 | 3 | 3 | 2 | 3 |
| Hawke, LD et al. | 5 | 2 | 3 | 3 | 3 | 1 | 2 | 3 |
| Stevens, V et al. | 5 | 2 | 3 | 3 | 3 | 1 | 1 | 3 |
| Jones, N et al. | 3 | 2 | 2 | 1 | 3 | 1 | 3 | 3 |
| Jones S, et al. | 7 | 3 | 2 | 3 | 3 | 3 | 3 | 3 |
| Kassam, A et al. | 2 | 1 | 3 | 1 | 2 | 1 | 3 | 3 |
| Kimber, B. | 7 | 3 | 2 | 3 | 3 | 2 | 1 | 3 |
| King, KA et al. | 5 | 2 | 2 | 3 | 3 | 1 | 3 | 3 |
| Kalafat, J & Elias, M | 2 | 1 | 2 | 1 | 3 | 2 | 3 | 3 |
| Mora M, et al. | 2 | 1 | 2 | 1 | 3 | 1 | 2 | 2 |
| Pervanas, HC et al. | 5 | 2 | 3 | 3 | 3 | 3 | 3 | 3 |
| Jerome, lW. | 5 | 2 | 2 | 3 | 3 | 3 | 3 | 3 |
| Kusel, AB. | 1 | 1 | 2 | 3 | 3 | 3 | 3 | 3 |
| **Author(s)** | **Study Design** | **Rating for study quality** | **Rating for selection bias** | **Rating for confounders** | **Rating of studies on blinding** | **Rating of studies on data collection** | **Rating of studies on withdrawals and drop-outs** | **Global rating** |
| Reis, J et al. | 3 | 2 | 2 | 3 | 3 | 3 | 3 | 3 |
| Roberts, G | 5 | 2 | 3 | 1 | 3 | 2 | 2 | 3 |
| Roberts, LM | 1 | 1 | 1 | 3 | 3 | 1 | 1 | 3 |
| Safer | 3 | 2 | 2 | 1 | 3 | 1 | 3 | 3 |
| Altindag A, et al. | 3 | 2 | 2 | 3 | 3 | 2 | 2 | 3 |
| Friedrich, B et al. | 2 | 1 | 2 | 1 | 3 | 1 | 3 | 3 |
| Kerby J, et al. | 2 | 1 | 3 | 1 | 2 | 1 | 3 | 3 |
| Tucker, JB et al. | 7 | 3 | 3 | 3 | 3 | 3 | 1 | 3 |
| Twardzicki, M et al. | 5 | 2 | 3 | 3 | 3 | 3 | 3 | 3 |
| Van Schoiack-Edstrom, L et al. | 3 | 2 | 2 | 1 | 3 | 2 | 3 | 3 |
| Essler, V et al. | 5 | 2 | 3 | 3 | 3 | 2 | 3 | 3 |
| Woods, DW and Marcks, BA | 2 | 1 | 1 | 1 | 3 | 1 | 1 | 2 |
| Woodside, M et al. | 3 | 2 | 3 | 1 | 3 | 3 | 3 | 3 |
| Rabak-Wagener, J et al. | 3 | 2 | 2 | 1 | 3 | 1 | 3 | 3 |
| Irving, LM & Berel, SR | 1 | 1 | 3 | 1 | 3 | 1 | 1 | 3 |
| Watson, R &Vaughn, LM | 3 | 2 | 3 | 1 | 3 | 1 | 3 | 3 |
| Chan, JY, Mak WW, Law LS. | 2 | 1 | 2 | 1 | 3 | 1 | 3 | 3 |
| Stuart, H. | 5 | 2 | 2 | 1 | 3 | 2 | 3 | 3 |
| Fernandez A, et al. | 1 | 1 | 2 | 3 | 1 | 1 | 1 | 2 |
| Pitre, N et al., | 2 | 1 | 3 | 1 | 1 | 2 | 1 | 2 |
| Conrad, I. et al. | 5 | 3 | 3 | 3 | 2 | 2 | 2 | 3 |
| **Author(s)** | **Study Design** | **Rating for study quality** | **Rating for selection bias** | **Rating for confounders** | **Rating of studies on blinding** | **Rating of studies on data collection** | **Rating of studies on withdrawals and drop-outs** | **Global rating** |
| Hui, C.L.M. et al. | 5 | 3 | 3 | 3 | 2 | 1 | 3 | 3 |
| Koike, et al. | 1 | 1 | 2 | 2 | 2 | 1 | 2 | 2 |
| \| **LEGEND** \| \| \| \| \| --- \| --- \| --- \| --- \| \| Component ratings \| 1=Strong \| 2=Moderate \| 3=Weak \| \| Study design \| 1= Randomised controlled trial; 2=controlled clinical trial; 3=cohort analytic (two group pre and post); 4= case control; 5=cohort (one group pre and post); 6=interrupted time series; 7=post-test only for one grp) \| \| \| | | | | | | | | |
