# Supplementary figures and images for: Effectiveness of arts interventions to reduce mental-health-related stigma among youth: a systematic review and meta-analysis
Source: BMC Psychiatry. 2021 Jul 22;21:364. doi: 10.1186/s12888-021-03350-8 (PMC8296649; doi:10.1186/s12888-021-03350-8)

**Single session**

**Knowledge**


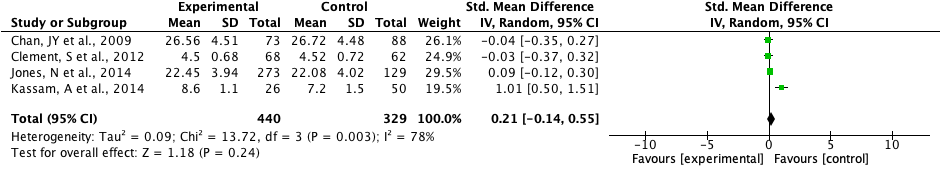


**Attitude**


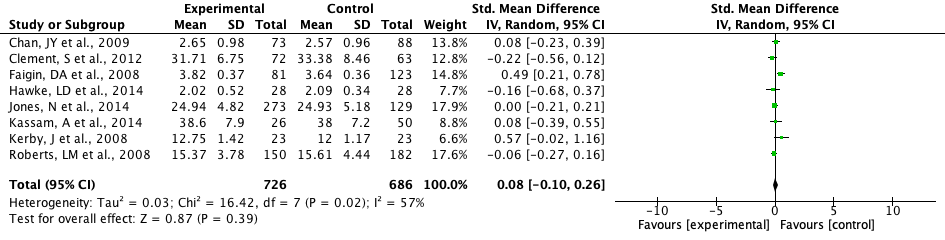


**Behavior**


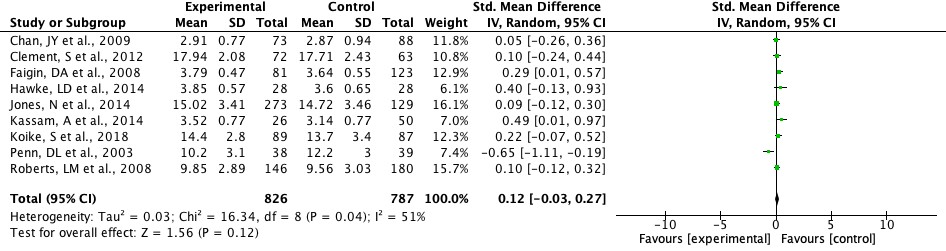
**Multi-session**

**Knowledge**


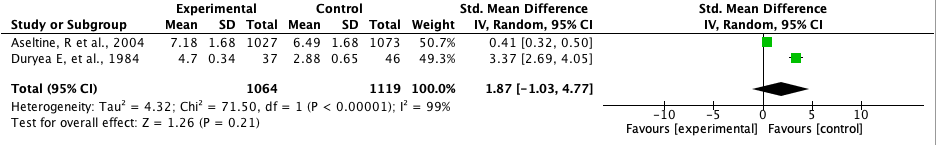


**Attitude**


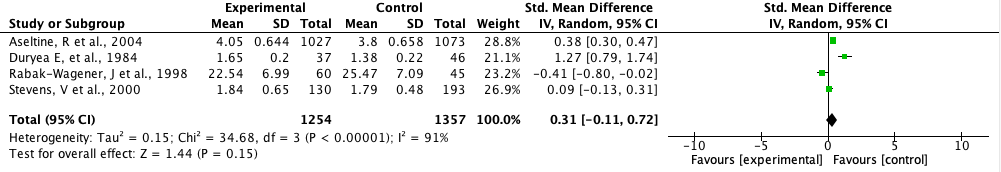


**Behavior**


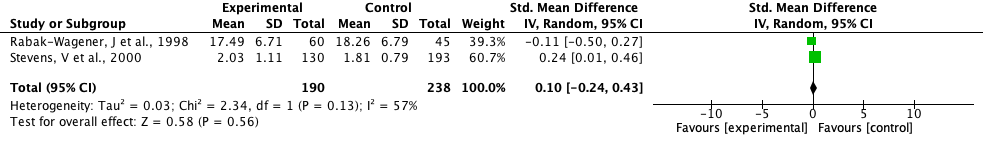

Supplement: Supplementary file 4 — Additional file 4. Meta-analyses of studies focusing on all KAB aspects of stigma (a comprehensive approach to measurement and possibly intervention.content). [file 12888_2021_3350_MOESM4_ESM.docx]
